# From Mediterranean Fever to Autoimmune Hepatitis

**DOI:** 10.7759/cureus.89896

**Published:** 2025-08-12

**Authors:** Inês Salvado de Carvalho, Maria João Baldo

**Affiliations:** 1 Internal Medicine, Unidade de Saúde Local da Guarda, Guarda, PRT; 2 Internal Medicine, Hospital Sousa Martins, Unidade de Saúde Local da Guarda, Guarda, PRT

**Keywords:** autoantibodies, elevated transaminases, hepatitis autoimmune, immune-mediated disease, rickettsia infections

## Abstract

Autoimmune hepatitis (AIH) is a chronic inflammatory liver disease of unknown etiology. It is a rare immune-mediated pathology without a typical clinical presentation and with a challenging diagnosis. The AIH has two types (types 1 and 2) that differ by the pattern of autoantibodies presented. Treatment is based on corticosteroid therapy with prednisolone, with or without azathioprine. The treatment aims to induce and maintain histological remission, improving symptoms and survival. Here, we present the case of a 72-year-old female patient admitted to the Internal Medicine Department with suspected toxic hepatitis associated with the use of cefuroxime. During hospitalization, due to lack of improvement after antibiotic discontinuation, an extensive etiological study was performed, with a diagnosis of type 1 AIH. After the histological diagnosis, she started the treatment with corticosteroids with a good therapeutic response.

## Introduction

Autoimmune hepatitis (AIH) is a chronic inflammatory liver disease characterized by increased transaminases and immunoglobulin G (IgG), the presence of autoantibodies, and histologically with interface hepatitis [1].

The etiology of AIH is still unknown, with a complex pathophysiology that requires interplay among genetic, epigenetic, immunological, and environmental factors. AIH is a challenging immune-mediated disease, as it does not have a typical presentation and can manifest clinically in a variable way, from an asymptomatic form with intermittent elevation of transaminases to fulminant hepatitis with acute liver failure. The disease is classified into at least two distinct types according to the nature of autoantibodies (types 1 and 2). The treatment of AIH is supported by immunosuppressive therapy. In the absence of treatment, this pathology can progress to cirrhosis, liver failure, and death [2].

It is a rare nosological entity, with a prevalence of 20 cases per 100,000 inhabitants [3]. AIH can affect individuals of any age, sex, ethnicity, and region. Some studies suggest that its incidence has increased, especially in elderly patients [4].

AIH is a diagnosis of exclusion, and there is no diagnostic test that can confirm it, which makes it difficult to approach these patients.

## Case presentation

A 72-year-old woman, an independent in activities of daily living, living in a rural region with frequent contact with animals (chickens, rabbits, roosters and hunting dogs), goes to the emergency department (ED) for pruritus, edema of the lower limbs and a feeling of increased abdominal circumference that began two days ago after starting to take antibiotics. The patient reported going to the health center two days ago for abdominal pain in the suprapubic region, mild type, grade 5 out of 10, without relief or aggravation factors, and accompanied by dysuria, choluria, and fetid urine. She underwent a urine test strip and was diagnosed with a urinary tract infection and medicated with cefuroxime. In addition, the patient reported asthenia, anorexia, weight loss, and sporadic sweating after two weeks of evolution, but without fever. He denied other symptoms, such as myalgias, headaches, nausea, vomiting, diarrhea or constipation, and respiratory and cardiovascular symptoms.

Of the relevant personal history, the following stand out: arterial hypertension; dyslipidemia; post-cholecystectomy status; and chronic venous insufficiency, medicated with perindopril + amlodipine + rosuvastatin (5 mg + 5 mg + 20 mg), one pill at breakfast. The patient reported regular alcohol consumption (two glasses of wine/day), no history of smoking, illicit drug use, and recent travel.

On objective examination, the patient was hemodynamically stable, apyretic with mucocutaneous jaundice, globose abdomen, with bowel sounds present and normal, soft and depressible timbre, painless to palpation, without palpable masses or organomegalia, without signs of peritoneal irritation, and mild edema of the lower limbs. To highlight the identification of a suspicious lesion of inoculation in the dorsal region, which the patient did not initially mention, but when questioned, he identified it as an arthropod bite, about two weeks ago, without being able to specify which. The results of the analytical tests revealed normocytic normochromic anemia, thrombocytopenia with prolonged clotting times, hyponatremia, hypoproteinemia with hypoalbuminemia, hepatic cytocholestasis, and hyperbilirubinemia (Table 1).

**Table 1:** Analytical evolution of the patient ALT - alanine aminotransferase, AST - aspartate aminotransferase, GGT - gamma-glutamyl transpeptidase, INR - international normalized ratio

Analytical values	On admission	After one month	Reference values
Hemoglobin (g/dL)	10.8	14.2	12-16
Platelets (10^3^/μL)	122	126	140-400
INR	2.07	1.16	-
AST (U/L)	949	65	5-34
ALT (U/L)	653	83	<34
Alkaline phosphatase (U/L)	268	188	46-122
GGT (U/L)	126	182	0-38
Total bilirubin (mg/dL)	5.68	1.54	0-0.5
Albumin (g/dL)	2.30	0.63	3.2-4.6
Ferritin (ng/mL)	1817.1	512.4	4.6-204
Unsaturated iron-binding capacity (ug/mL)	< 25	-	70-310
Transferrin (mg/mL)	117	-	173-204
Iron (ug/mL)	162	-	50-170
Folic acid (ng/mL)	9.7	-	3.1-20.5
Vitamin B12 (pg/mL)	1033	-	187-883
IgG Immunoglobulin (mg/mL)	2506.1	1394.2	610.3-1616

**Figure 1:**
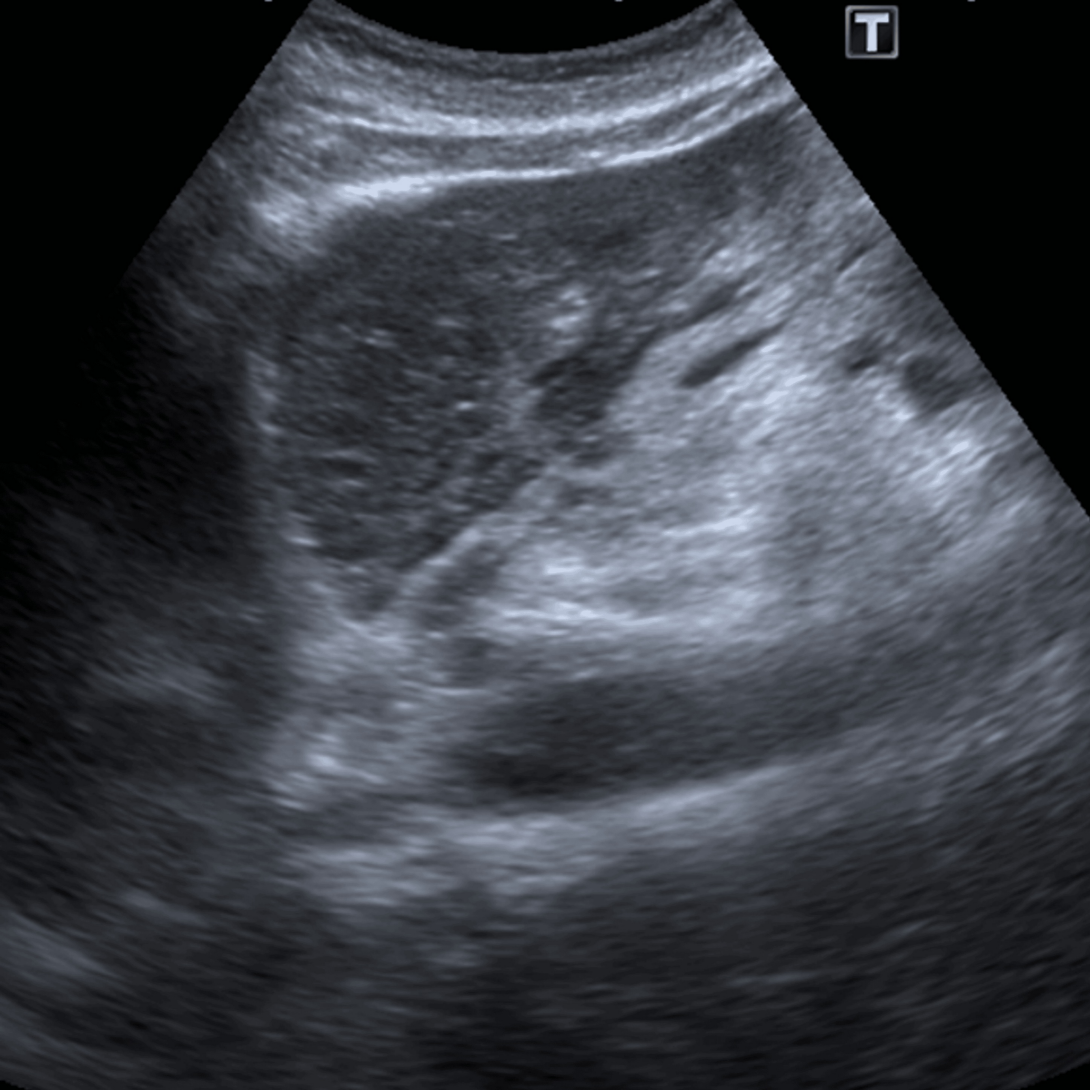
Abdominal ultrasound image of the patient’s globose liver

**Figure 2:**
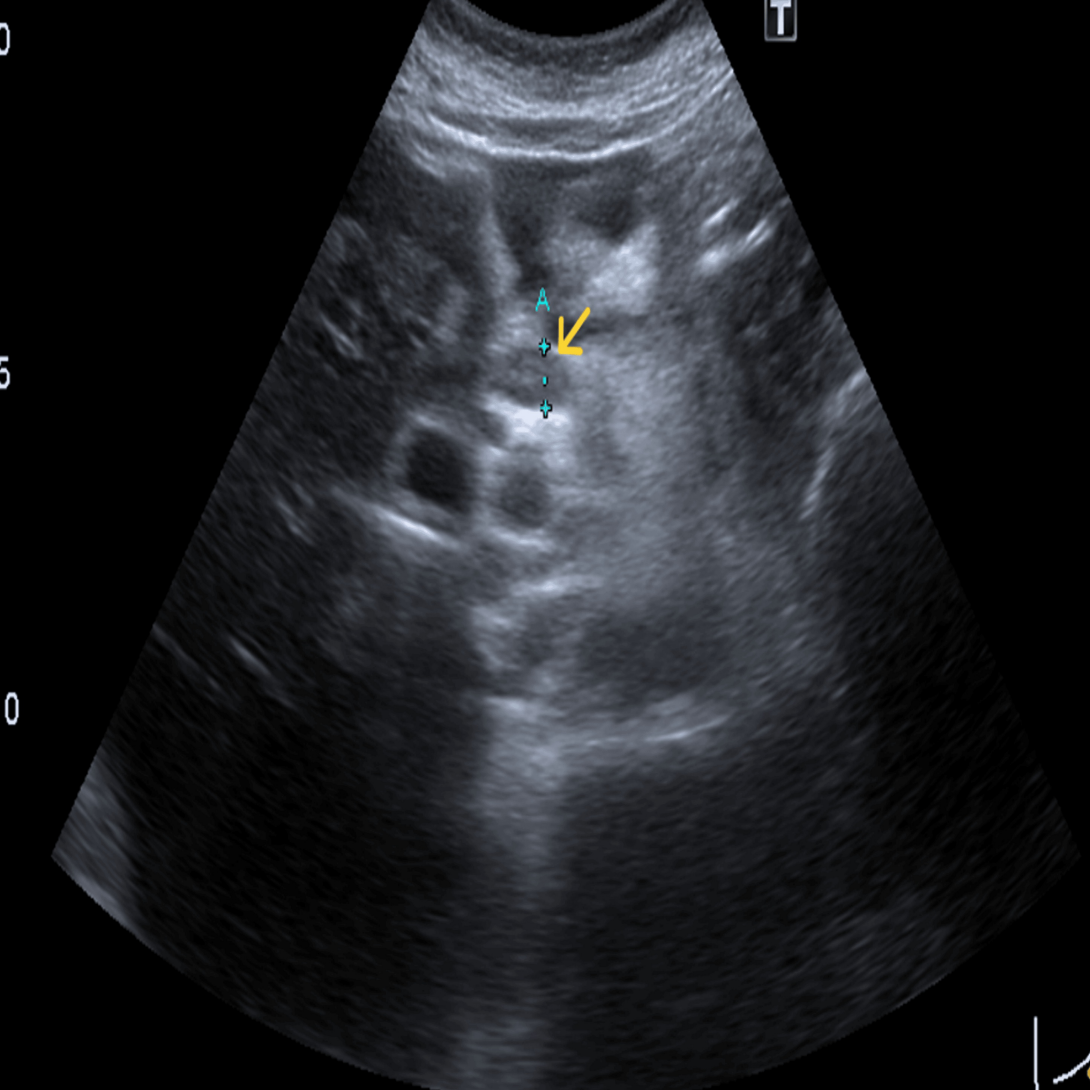
Abdominal ultrasound of hepatic ganglia at the level of the hepatic hilum

**Figure 3:**
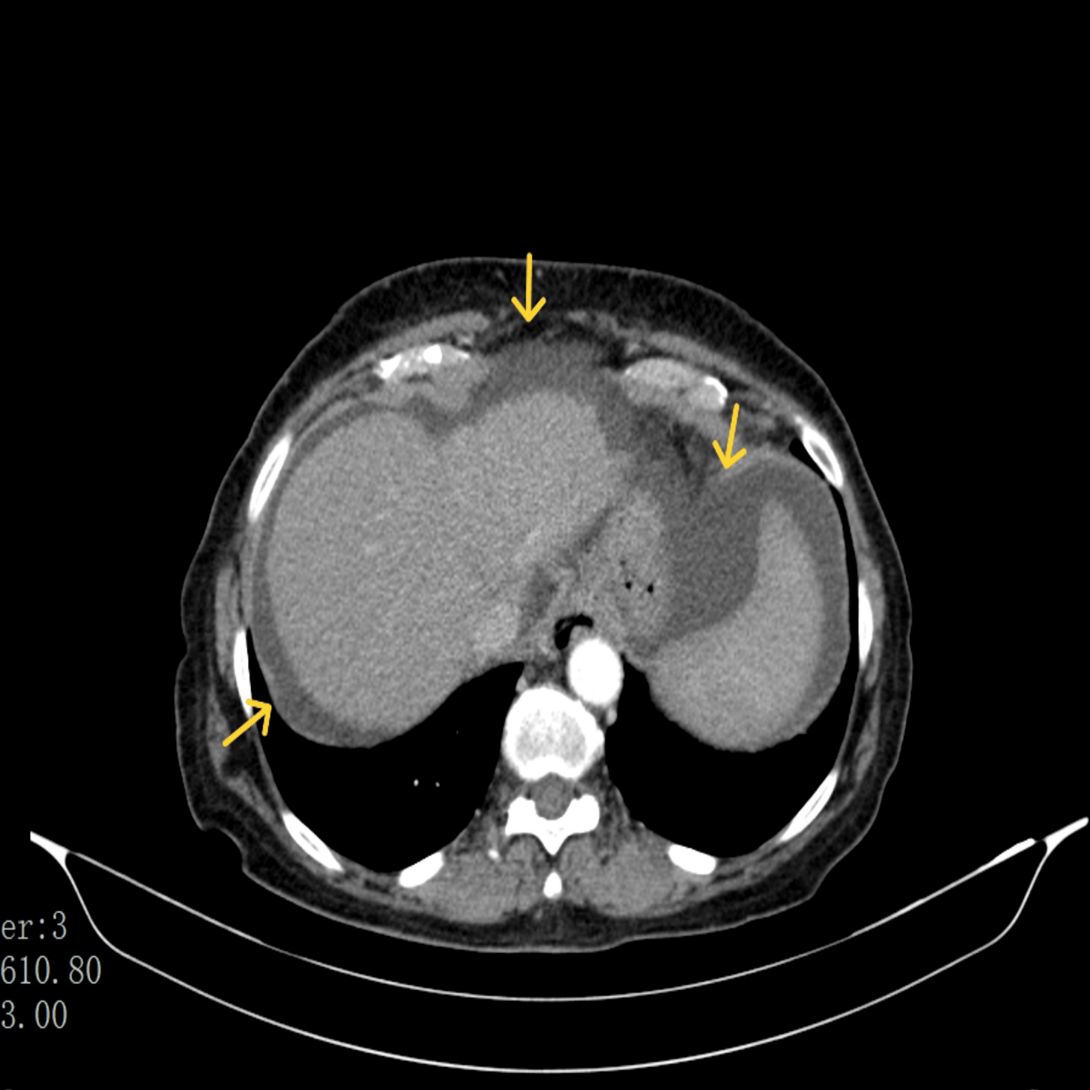
Abdomino-pelvic CT scan with scattered ascites

In this context, an abdominal ultrasound was performed and identified a globose liver, particularly in the lateral segments of the left lobe, with a slightly heterogenous structural pattern (Figure 1), several nonspecific hepatic ganglia at the level of the hepatic hilum of 9 mm with a short axis (Figure 2) and with small to moderate peritoneal effusion. An abdomino-pelvic CT scan was performed for better clarification, which revealed scattered ascites (Figure 3).

Due to suspicion of toxic hepatitis associated with antibiotic therapy, the patient was hospitalized with suspension of cefuroxime administration and with a request for a complementary study to exclude other possible toxic and infectious causes.

From the complementary study, hepatotropic viral markers were negative and, consequently, infections by Epstein-Barr virus, Cytomegalovirus, Parvovirus B19, Brucella, and Leptospira were ruled out. Considering the clinical and epidemiological context, accompanied by a positive Weil-Felix reaction (anti-Proteus antibody OX19 > 320), Mediterranean fever was assumed, and doxycycline 100 mg (12/12 hours) was started for seven days. After one week of hospitalization, the patient maintained pruritus and jaundice, with improvement of the remaining symptoms. Analytically, the patient presented a progressive worsening of thrombocytopenia with the appearance of petechiae, which were predominantly in the lower limbs. In view of a limited clinical and analytical improvement in the absence of possible toxicants and with a resolved infectious picture, other possible causes of liver diseases were analyzed.

The values of ceruloplasmin and alpha1 antitrypsin were within normal values. Of the hematinic factors, increased ferritin (9× upper limit of normality (ULN)), decreased unsaturated iron and transferrin binding capacity, and slightly elevated vitamin B12 levels (Table 1) were highlighted. Magnetic resonance imaging was performed, which ruled out hepatic iron overload and identified the liver with heterogeneous bosselated contours, suggestive of chronic liver disease. Protein electrophoresis showed the presence of a monoclonal IgG component, kappa, with an increased IgG titer (1.5× ULN), but with normal values for the remaining immunoglobulins. For better elucidation, a medulogram was performed, which showed aspects compatible with anemia of chronic diseases and immunophenotyping with findings suggestive of reactive erythropoiesis and neutropoiesis. From the autoimmune study, the patient had positive anti-thyroglobulin antibody, negative anti-peroxidase, and normal thyroid function. Of the remaining autoantibodies tested, anti-cellular antibodies (ANA), anti-smooth muscle actin (SMA) (RC5), and doubtful anti-dsDNA antibody were positive (Table 2).

**Table 2:** Serology and antibodies Ac. - antibody; ANA - antinuclear antibody; ANCA - anti-neutrophil cytoplasmic antibody; HAV - hepatitis A virus; HBV - hepatitis B virus; HCV - hepatitis C virus; HIV - human immunodeficiency virus; SMA - smooth muscle actin

Further study	Result
Viral serology (HCV, HBV, HAV, HIV)	Negative
Weil-Felix reaction (anti-Proteus antibody OX19)	> 320 (Positive)
Ac. anti-thyroglobulin	++
Ac. anti-peroxidase	Negative
ANA	++
HIP	Negative
SMA (actin)	+++
Ac. soluble hepatic antigen/pancreatic liver (SLA/LP)	Negative
Ac. anti-liver and kidney microsomal 1 or 3 (LKM1/3)	Negative
Ac. anti-hepatic cytosol type 1 (LC1)	Negative
Ac. anti dsDNA	Doubtful

In view of the diagnostic doubt and given the positive hepatic autoimmunity (smooth muscle actin antibody and antinuclear antibody) with increased IgG, the hypothesis of AIH was raised, and the patient underwent liver biopsy. Histologically, interface hepatitis was observed, and at the periseptal lobular level, some outline of rosettes of the hepatocytes was verified, confirming the anatomical pathological diagnosis of AIH with moderate activity, in the cirrhotic phase.

Treatment with corticosteroid therapy is initiated with prednisolone 40 mg, resulting in a favorable clinical and analytical evolution, with resolution of pruritus and jaundice, improvement in liver function, platelets, and hemoglobin within two weeks (Table 1).

## Discussion

AIH is a chronic inflammatory liver disease. The diagnosis is based on the presence of a set of compatible features (histological, clinical, and laboratory) and exclusion of possible etiologies of liver disease, such as viral (hepatitis B and C), alcoholic, hereditary (hemochromatosis, Wilson's disease, alpha-1 antitrypsin deficiency), cholestatic (autoimmune cholangitis, primary biliary cirrhosis), and drug-induced.

AIH is divided into two types, which are differentiated by the pattern of autoantibodies present. Type 1 is characterized by the presence of ANA, SMA, anti-actin and/or anti-SLA/LP antibodies, and the latter, despite being present in only 10-20% of cases, have a high specificity for diagnosis. AIH type 2 is defined by the positivity of anti-liver and kidney microsomal fraction 1 or 3 (LKM1/3) and/or anti-hepatic cytosol type 1 (LC1) antibodies [5]. In some cases, the patient may have all the hallmarks of AIH and respond to treatment, but without characteristic circulating antibodies, which defines seronegative AIH. Clinically, it can present in a variety of ways, which makes diagnosis difficult. Most patients present nonspecific symptoms of chronic evolution, such as asthenia, general malaise, arthralgias, anorexia, weight loss, amenorrhea, and nausea. They may present with signs of chronic liver disease, such as ascites, palmar erythema, jellyfish head abdomen, and manifestations of extrahepatic autoimmune disease. Other extrahepatic autoimmune diseases may be associated, present in 14-44% of AIH cases, with autoimmune thyroid disease being the most frequent [6]. This pathology is characterized by an elevation of transaminases, which can reach values 12 to 50 times the ULN, while cholestatic enzymes can be normal or slightly increased. Typically, with hypergammaglobulinemia, there are remaining normal immunoglobulins and the presence of one or more characteristic autoantibodies [7]. The onset of AIH may be more acute (course less than 30 days), with acute liver failure, jaundice, and hepatic encephalopathy. In these cases, it is important to exclude whether it is a spontaneous exacerbation or triggered by a trigger, whether infection or toxic, in an undiagnosed AIH. In fact, the patient in the exposed case presents clinical findings (nonspecific symptoms, jaundice, and ascites with evolution < 30 days) and analytical findings (elevation of transaminases, prolongation of coagulation time, and hypergammaglobulinemia) that is possible to be compatible with an exacerbation of an undiagnosed AIH, with *Rickettsia* infection. Associated with positive ANA and SMA antibodies, which fits into a type 1 AIH, the most frequent typology.

The diagnosis of AIH cannot be made without compatible histological features, such as hepatitis interface with lymphoplasmacytic infiltrate, emperipolesis, hepatocytes in rosettes, hepatocyte edema, and pyknosis [4,8]. The coexistence of biliary alterations, such as bile duct injury, guides the diagnosis to an "overlapping syndrome" of AIH and primary biliary cholangitis. In the clinical case presented, liver biopsy identified the typical characteristics of AIH, but with findings of cirrhosis at the time of diagnosis, which occurs in one third of the patients, especially with advanced age, as shown in the case presented.

In 1993, the International Group for the Study of Autoimmune Hepatitis created a scoring system with diagnostic criteria, which was later revised and simplified [9]. Although used essentially in the context of research, the original score and the simplified score are available in clinical practice, which aim to assist in the diagnosis of AIH in complex clinical cases; however, they can only be used after performing a liver biopsy. In the clinical case exposed, in view of the clinical, analytical, and histological context, the simplified AIH score has a result of seven points, which is considered a definitive diagnosis of AIH.

The treatment of AIH is based on corticosteroid therapy, prednisone/prednisolone, which may or may not be associated with azathioprine. It has been proven that treatment with corticosteroids induces and maintains histological remission, improves symptoms, and increases survival. Azathioprine is especially used to maintain laboratory remission, allowing lower doses of corticosteroids, in order to reduce its adverse effects such as weight gain and osteoporosis with long-term use. Rapid response to treatment (improvement in transaminase levels within two weeks) is one of the best predictors of prognosis. The guidelines suggest that a reduction in therapy after two years with sustained normal levels of AST, ALT, and IgG, considered criteria for remission, should be considered. However, although most patients achieve remission, about 46% of adults relapse when medication is suspended or decreased [10]. The patient portrayed here started treatment with prednisolone monotherapy, and in two weeks of treatment, she showed clinical improvement and a significant reduction in AST and ALT, which is considered a good predictor of prognosis. 

The survival of patients with treated AIH, in recent studies, was 91% at 10 years [2]. However, the presence of cirrhosis at the time of diagnosis is associated with reduced survival.

## Conclusions

AIH is a well-defined syndrome that does not have any specific diagnostic marker. An immune-mediated disease whose pathophysiology is not fully understood. It presents in a variable manner (insidious, acute, or even fulminant) and with poor clinical presentation, often making diagnosis difficult.

A definitive diagnosis of AIH can only be made with the presence of compatible histopathological findings obtained by liver biopsy. The identified immunoserological pattern allows us to distinguish AIH into types 1 and 2, although in a minority of cases, AIH may occur without an identified immunoserological pattern. Treatment is based on immunosuppression, often with a rapid response. Although most patients achieve remission, relapses are common when immunosuppression is discontinued. Untreated AIH can lead to more serious complications, such as cirrhosis, liver failure, and death.

## References

[1] Wang QX, Yan L, Ma X (2018). Autoimmune hepatitis in the Asia-Pacific area. J Clin Transl Hepatol.

[2] Mack CL, Adams D, Assis DN (2020). Diagnosis and management of autoimmune hepatitis in adults and children: 2019 practice guidance and guidelines from the American Association for the Study of Liver Diseases. Hepatology.

[3] Lv T, Li M, Zeng N (2019). Systematic review and meta-analysis on the incidence and prevalence of autoimmune hepatitis in Asian, European, and American population. J Gastroenterol Hepatol.

[4] Heneghan MA, Lohse AW (2025). Update in clinical science: autoimmune hepatitis. J Hepatol.

[5] European Association for the Study of the Liver (2015). EASL Clinical Practice Guidelines: autoimmune hepatitis. J Hepatol.

[6] Bittencourt PL, Farias AQ, Porta G (2008). Frequency of concurrent autoimmune disorders in patients with autoimmune hepatitis: effect of age, gender, and genetic background. J Clin Gastroenterol.

[7] Manns MP, Czaja AJ, Gorham JD, Krawitt EL, Mieli-Vergani G, Vergani D, Vierling JM (2010). Diagnosis and management of autoimmune hepatitis. Hepatology.

[8] Czaja AJ, Carpenter HA (1993). Sensitivity, specificity, and predictability of biopsy interpretations in chronic hepatitis. Gastroenterology.

[9] Czaja AJ (2008). Performance parameters of the diagnostic scoring systems for autoimmune hepatitis. Hepatology.

[10] van Gerven NM, Verwer BJ, Witte BI (2013). Relapse is almost universal after withdrawal of immunosuppressive medication in patients with autoimmune hepatitis in remission. J Hepatol.

